# Patient and staff experiences with an EHR-Integrated Symptom Management Program (eSyM) in oncology

**DOI:** 10.1007/s00520-025-10248-8

**Published:** 2025-12-24

**Authors:** Christine M. Cronin, Fiona Barrett, Samira Dias, Roshan Paudel, Raymond U. Osarogiagbon, Deb Schrag, Sandra L. Wong, Don S. Dizon, Jessica J. Bian, Hannah W. Hazard-Jenkins, Gabriel A. Brooks, Anna Revette, Michael J. Hassett

**Affiliations:** 1 Dana-Farber Cancer Institute, 450 Brookline Avenue, Boston, MA 02215 USA; 2 Multidisciplinary Thoracic Oncology Program, Baptist Cancer Center, Memphis, TN USA; 3 Memorial Sloan Kettering Cancer Center, New York, NY USA; 4 Emory University, Atlanta, GA USA; 5 Tufts Medical Center, Boston, MA USA; 6 MaineHealth Cancer Care, South Portland, ME USA; 7 West Virginia University Cancer Institute, West Virginia University, Morgantown, WV USA; 8 Dartmouth Hitchcock Medical Center, Lebanon, NH USA

**Keywords:** Qualitative interviews, EPROs (electronic patient-reported outcomes), EPRO-based symptom management, ESyM (electronic symptom management), Oncology, Implementation science

## Abstract

**Context:**

To bolster integration of electronic patient-reported outcomes (ePROs) into routine practice, a consortium of six cancer centers developed an EHR-integrated electronic symptom management program (eSyM) and deployed it in an institutional randomized stepped-wedge hybrid effectiveness-implementation study involving patients receiving chemotherapy or surgery.

**Objectives:**

We conducted qualitative interviews with patients and staff after implementing eSyM to understand and compare experiences and perceptions regarding the intervention and barriers/facilitators to ePRO usage in routine oncology practice.

**Methods:**

We developed semi-structured interview guides for patients and both clinical and non-clinical staff exposed to eSyM. Questions were informed by the Consolidated Framework for Implementation Research (CFIR). Patients provided consent; a consent waiver was obtained for staff. Interview transcripts were dual-coded and thematically analyzed.

**Results:**

From September 2020 to March 2024, 79 staff and 14 patients participated in interviews. Staff and patients held similar beliefs regarding eSyM compatibility, communication, and prioritization. Most perceived that eSyM was easy to use and improved communication, but competing demands impeded regular utilization, and deeper engagement was necessary for sustainability. Discordance emerged in the domains of receptivity and technology. Patients were more receptive to eSyM and to using technology than staff perceived.

**Conclusion:**

Most patients and staff reported that eSyM added value and was easy to navigate. Staff tended to underestimate patients’ receptivity to eSyM. De-prioritization of eSyM could reflect a lack of perceived mutual engagement in or impact from the intervention. Understanding perceived and experienced facilitators and barriers can enable successful implementation of ePRO systems like eSyM to improve cancer care delivery.

**Supplementary Information:**

The online version contains supplementary material available at 10.1007/s00520-025-10248-8.

## Introduction

Electronic patient-reported outcomes (ePROs) capture patients’ perceptions of well-being and symptom burden without clinician amendments or interpretations [1–4]. Dissemination of electronic health records with patient portals supports ePRO use, enabling longitudinal monitoring of treatment-related side effects and functional status changes throughout cancer treatment and survivorship [5]. Clinical trials have shown that collecting ePROs reduces symptom burden and acute care needs [6, 7], improving overall survival amongst patients with cancer [8]. Interest in integrating ePROs into routine oncology practice is increasing, bolstered by guidelines from the Centers for Medicare and Medicaid requiring sites participating in the Enhancing Oncology Model (EOM) to implement ePROs to qualify for reimbursement [9, 10].

As demand for robust, easily implementable ePRO programs increased, the Symptom Management Implementation of Patient-Reported Outcomes in Oncology (SIMPRO) Research Consortium, a group of six diverse health systems, partnered with Epic™, the EHR (electronic health record) vendor, to develop and deploy an electronic symptom management program (eSyM) into the standard clinical workflow [11, 12]. eSyM is an intervention that enables patients to report, and care teams to triage, symptoms in real-time. At each SIMPRO site, eSyM was available through routine care for all patients in participating clinics with a suspected or confirmed gastrointestinal, colorectal, gynecological, or thoracic cancer who were receiving chemotherapy or surgery. Patients complete 12 PRO-CTCAE-based symptom items and two health-related quality of life items up to three times per week [11, 13].

One major goal of the SIMPRO Research Consortium was to address hurdles associated with ePRO adoption by creating an implementation-ready, EHR-integrated tool for ePRO reporting. eSyM is now available to all Epic-enabled institutions, helping to fulfill this goal [14]. This approach was used because prior stakeholder feedback revealed that, without integration into routine workflows, ePRO reports would be underutilized and increase staff burden. Deploying an integrated eSyM solution is only the first step, however. Optimal program uptake requires robust patient and staff engagement. Without effective strategies and sustained support, the potential clinical and quality of life benefits associated with ePRO usage cannot be fully realized.

It is critical to understand barriers and facilitators to eSyM use to build off the successful integration of eSyM and ensure utilization. During the roll-out across SIMPRO’s six health systems, a series of studies were conducted to comprehensively assess the acceptability of the intervention and its implementation. Here, we report findings from semi-structured qualitative interviews to understand commonalities and differences in patient and staff perspectives on the eSyM implementation process.

## Methods

SIMPRO sites (Baptist Cancer Center, Dana-Farber Cancer Institute, Dartmouth-Hitchcock Medical Center, Lifespan Cancer Institute (now Brown University Health Cancer Institute), Maine Medical Center, and West Virginia University Cancer Institute) developed and deployed eSyM from 2018 to2023 through a hybrid effectiveness-implementation study (Clinicaltrials.gov study #: NCT03850912; initial registration date: 2/20/2019) [11, 13], in partnership with the Improving the Management of symPtoms during And following Cancer Treatment (IMPACT) Consortium, an NCI-funded research initiative focused on improving symptom control through EHR-integrated ePROs for systematic reporting and guideline-based clinical management [15]. These methods adhere to COREQ standards (Supplementary Material 1).

### Population identification and interview guide development

We identified three target subgroups for qualitative data collection — patients, clinical staff, and operational staff who had interacted with eSyM. These high-level subgroups were prioritized because eSyM had distinct features across subgroups with little variation in program exposure within subgroups. Participants were identified through purposeful sampling.

Semi-structured interview guides were developed iteratively to introduce the project, explain reasons for conducting the research, and present questions about the interviewees’ experiences with eSyM (Supplementary Materials 2– 3). Interview questions were informed by literature review, the Consolidated Framework for Implementation Research (CFIR), and recommendations from qualitative research experts [16]. Study investigators tailored the interview guide to the features of the SIMPRO sites and eSyM intervention, while ensuring that each CFIR domain (innovation, outer setting, inner setting, individuals, and process) was addressed [17]. Table 1 shows how the interview questions were mapped to CFIR constructs (Table 1). The interview guide was also tailored for the different subgroups to ensure that all relevant experiences were queried. Before being disseminated, the preliminary guide was reviewed by site investigators and qualitative research experts and revised accordingly. Additional details on the process of iterative development and adaptation have been previously reported [17].

**Table 1:** Interview questions mapped to select CFIR categories

CIFR Domain	CFIR Construct	Care Team Example Question	Patient Example Questions
Intervention Characteristics	*Evidence Strength*	What do your team members generally think about integrating ePROs into cancer care?	Do you feel that your care team was viewing and responding to your eSyM questionnaires?
	*Relative Advantage*	What is your evaluation of the value of eSyM at your site so far?	Overall, did you find the eSyM program helpful?
	*Adaptability*	What should be changed to make eSyM work better?	Are there any questions you would change in the questionnaire?
	*Complexity*	How complicated do you think eSyM is for patients to use?	Were you able to easily navigate the eSyM homepage and questionnaire?
	*Design Quality*	Have you reviewed the symptom management "tip sheets"?	Did you utilize the symptom management tip sheets?
Outer Settings	*Patient Needs and Resources*	What factors made it easier/more difficult for your patients to use eSyM?	What was your biggest barrier (if any) in using eSyM?
	*Opinion Leaders*	Who are the key leaders needed to make eSyM implementation successful at your site?	Was eSyM mentioned by your provider in appointments you had with them?
	*External Policies and Incentives*	Do you think COVID-19 will affect the effectiveness of eSyM?	Have you ever completed questionnaires for other medical appointments?
Inner Settings	*Implementation Climate*	What factors have to be in place to increase the chances that eSyM implementation will be a success?	What, if anything, would make you more likely to use a program like eSyM?
Individual Characteristics	*Knowledge and Beliefs About the Intervention*	Do you think eSyM is effective in helping patients manage their symptoms from home?	Do you feel that your symptom management improved with eSyM?
	*Self-Efficacy*	How confident are you that you are able to use eSyM?	Did you have assistance filling out the eSyM questionnaires?
Process	*Engaging*	What else could be done to engage patients?	Did eSyM impact your ability to communicate with your care team?
	*Executing*	What hasn’t worked well or could be improved?	Do you think the cadence at which you receive new eSyM questionnaires is appropriate?
	*Reflecting and Evaluating*	What does a site considering adopting eSyM need to know?	How would you describe your overall care experience? Did eSyM help/hinder that experience?

**Fig. 1:**
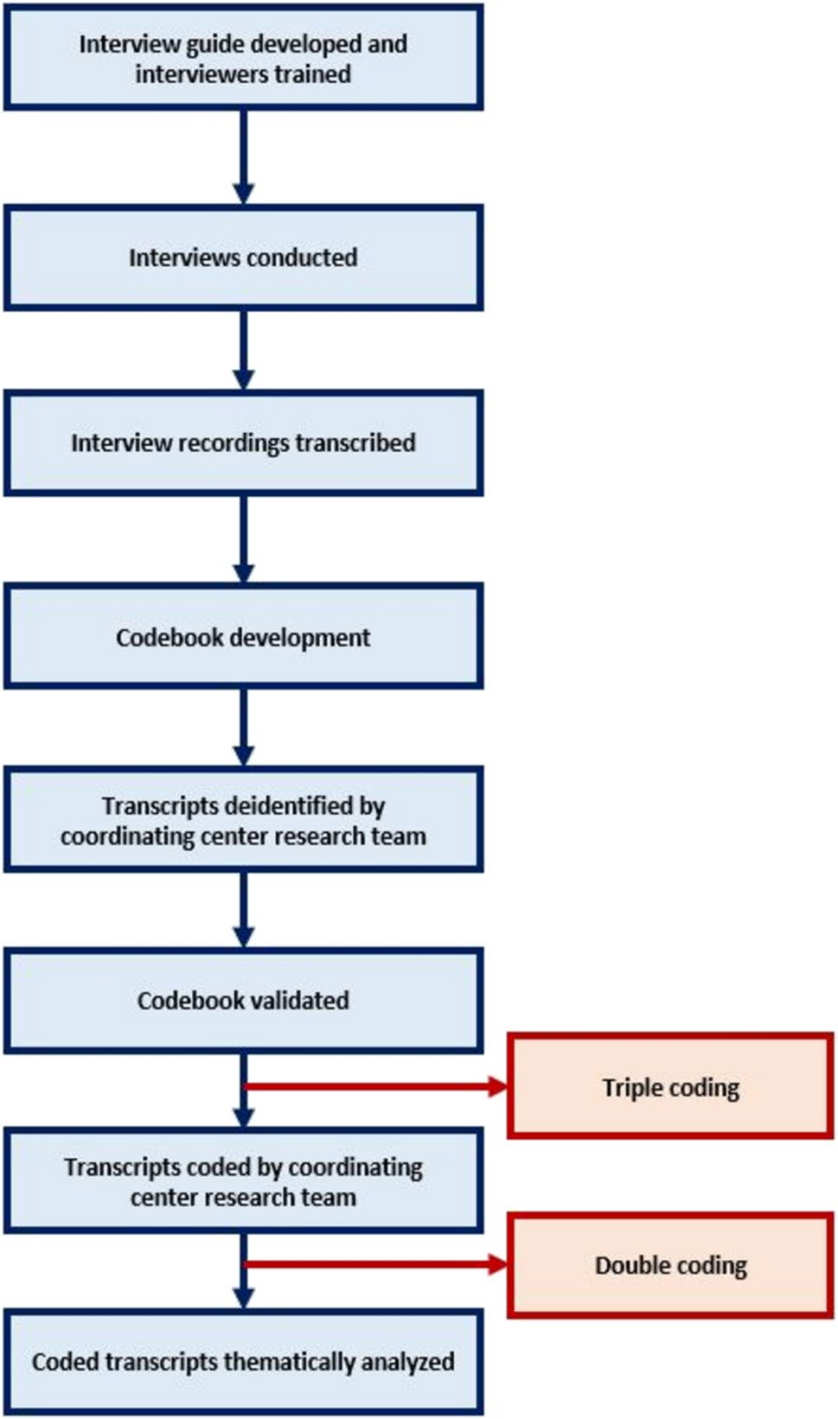
Overview of qualitative process. Accessibility Caption: Diagram showing the qualitative process. Interview guides were developed, and interviewers were trained. Interviews were then conducted, recorded, and transcribed. Transcriptions were deidentified. Codebooks for the transcripts were developed and validated. After validation, transcripts were triple- and then double-coded. The final codes were then thematically analyzed

Interviewers (DS, CC, FB) underwent training with Dana-Farber’s Survey and Qualitative Methods Core on proper interviewing methodology, techniques, and practices. Each interviewer had prior experience with qualitative data collection, ranging from 2 to >10 years. Interviewers included one principal investigator (DS), project manager (CC), and research assistant (FB) from the coordinating center. To ensure sufficient representation, staff and patient participants were recruited across SIMPRO sites (Fig. 1).

### Staff interviews

Staff were eligible to participate if they had used eSyM in routine clinical practice for a minimum of 12 months, emphasizing the recruitment of staff who had robust experience with the intervention. Site research teams identified appropriate staff through purposeful sampling and referred them to the coordinating center at Dana-Farber [18]. All sites sought to recruit a minimum of eight staff members: four each from surgical and medical oncology (minimum recruitment of 48 staff members SIMPRO-wide).

We conducted interviews between September 2020 and April 2023, timed to occur 12–15 months after eSyM implementation to allow for program stabilization and exposure. A waiver of informed consent was granted by the IRB of record (Western Internal Review Bureau) recognizing that staff interviewees were participating in a minimal-risk quality improvement project. Group interviews were conducted when multiple participants were available during a limited time frame and could not be interviewed separately. The 30–60 min interviews were conducted via Zoom or in-person based on participant preference. Interviewing ceased upon reaching meaning saturation, or the point where no further dimensions, nuances, or insights of issues could be found, as determined by the study team [19].

### Patient interviews

Patients were eligible if they submitted at least one eSyM questionnaire, were ≥18 years old, and had received treatment or surgery for a suspected or confirmed thoracic, gynecologic, or gastrointestinal cancer. Patients were identified by site teams and provided written consent. Emphasis was placed on speaking with patients who had used the eSyM program at least once, as they could provide feedback on the program’s acceptability and suggest enhancements; patients with no reported eSyM use were not eligible to participate. The pre-specified study plan proposed conducting patient interviews until thematic saturation was achieved or until 100 interviews were completed. At a minimum, each site sought to recruit four patients, two surgical and two medical oncology (minimum recruitment of 24 patients). Patients were invited to opt-in to participate through in-person, phone, and email approaches.

Interviews were conducted between September 2023 and March 2024, 30–180 days after a patient first used eSyM. Participants received a $15 gift card as renumeration. Interviews were conducted via Zoom or telephone, based on interviewee preference, and lasted 30–60 min. Persistent challenges with patient recruitment led the interviewers and study investigators to confer with the qualitative research expert overseeing this work (AR). They reviewed transcripts, conducted initial coding, and determined that meaning saturation had been achieved. Considering the challenges with patient recruitment, and that research operations were concluding as the grant period was coming to an end, interviewing ceased earlier than initially planned, but the sample was deemed sufficient for thematic analysis based on the preliminary analysis described above and considering that prior work has demonstrated that data saturation can be achieved with this sample size [20].

### Codebook development and thematic analysis

Interviews were recorded, transcribed, anonymized, and reviewed. A common analytic codebook was developed for all subgroups using interview guides, CFIR Framework, and emergent domains (Supplementary Materials 4). The codebook prioritized evaluation of facilitators and barriers to eSyM utilization, specifically regarding the intervention and its impact on clinical workflows and communication. Interview transcripts were coded using NVivo [21]. Coders (CC, FB, SD, AR) underwent training with Dana-Farber’s Survey and Qualitative Methods Core on proper coding techniques. Each coder had ≥2 years’ prior experience with qualitative data analysis. Coders included one qualitative research scientist (AR), two project managers (CC, SD), and one research assistant (FB) from the coordinating center. The first 25% of all interview transcripts were triple-coded to ensure methodologic consistency; subsequent transcripts were dual-coded. Meetings followed to review code selections and reach consensus, allowing the study team to assess different interpretations of code applications.

The study team employed a collaborative approach to codebook development and coding by including interdisciplinary voices and team discussions that brought diverse perspectives and expertise to the process. We prioritized team meetings to discuss the development of the codebook, application of the codes, and data interpretation as a meaningful way to create collaborative and engaged exploration of these data. Through these meetings, we were able to have a more comprehensive understanding of the codes, text, and emergent ideas. In this way, we utilized a team-based approach to coding and analysis enabling in-depth exploration of meaning and ensuring inclusiveness of perspectives.

Dual-coding was applied as a heuristic tool to facilitate team-based discussion and to examine cohesion between coders; a formal inter-rater reliability (IRR) calculation was not reported, in part due to the size of the patient sample [22]. Decisions regarding dual-coding were guided by the recommendations in the literature, which suggest that 10–25% of data is sufficient to apply multiple coding [23]. Triple coding 25% of the transcripts allowed for in-depth discussions regarding how codes were named, defined, and applied and enabled a breadth of perspectives to be included in the subsequent coding.

SIMPRO used an inductive approach for analyses, prioritizing the identification of thematic domains and commonalities between groups. Individual codes were summarized, and groupings were created based on prior ePRO qualitative research, CFIR framework, and expert input. Groupings were then analyzed to identify salient concepts, dynamics, and contexts within and across patient and staff groups, emphasizing the identification of commonalities and discordance in group experiences. Themes were developed and revised based on the convergence and divergence between groups.

## Results

**Fig. 2:**
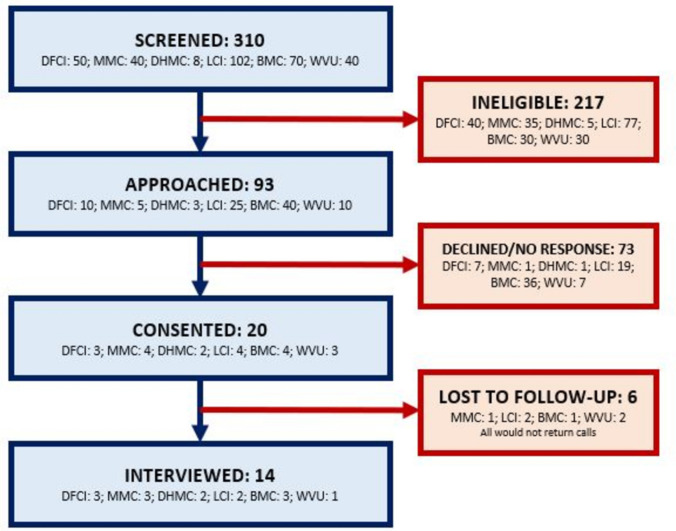
Patient interviews consort diagram. Accessibility caption: Consort diagram detailing the patient consenting process for interviews. 310 patients were screened across sites (210 deemed ineligible), 93 were approached (73 declined or did not respond), 20 were consented, and 14 were interviewed (6/20 would not return interviewer calls)

From September 2020 to March 2024, 79 staff members (three leadership, 22 MDs, 21 researchers, 26 nurses, three PAs, and four administrators) participated in individual interviews and group interviews (50 and 29, respectively), and 14 patients participated in interviews (Table 2). Staff members were invited to participate in interviews during existing team meetings and through direct invitation by a SIMPRO site coordinator, so the response rate for staff was not tracked; 15% of patient respondents agreed to participate (14/93) (Fig. 2) via the opt-in procedures used to recruit patients. Thematic analysis revealed that staff and patient interviewees held similar beliefs regarding eSyM workflow compatibility, impacts on patient-provider communication, and overall program prioritization. Discordance between patient and staff eSyM users emerged in the identified domains of post-symptom reporting expectations and receptivity to technology. Summary results with select quotes are described below; additional quotes for each thematic domain can be found in Supplementary Materials 5–6.

**Table 2:** Distribution of interviewees by role across SIMPRO sites

	Dana-Farber	West Virginia	Baptist	Lifespan	Maine	Dartmouth Hitchcock	SIMPRO Coordinating Center	Total
Leadership	0	0	1	1	1	0	0	**3**
Nurse	6	4	1	5	9	1	0	**26**
Research	0	2	5	2	1	1	10	**21**
Medical Doctor	8	2	0	2	2	8	0	**22**
Physician Assistant	2	0	0	1	0	0	0	**3**
Hospital Administrator	4	0	0	0	0	0	0	**4**
Surgical Patient	1	1	1	0	1	1	n/a	**5**
Medical Oncology Patient	2	0	2	2	2	1	n/a	**9**
Total	**23**	**9**	**10**	**13**	**16**	**12**	**10**	**93**

No staff participants declined to participate in interviews. Scheduling and availability limitations prevented 12 staff members from partaking in interviews

### Commonalities in patient and staff eSyM interactions

#### Workflow compatibility

Staff and patients expressed that eSyM was compatible with existing clinical workflows and technologic abilities. Staff interviews revealed consistent opinions that eSyM was minimally disruptive and well-integrated into clinical workflow, refuting pre-implementation concerns that eSyM symptom alerts would be burdensome. Care teams were not overwhelmed by responses, with most receiving a few notifications per week. Alerts were typically routed to nursing pools, so the responsibility to respond to ePRO symptoms could be shared among staff members and would be aligned with usual workflows regarding patients’ clinical concerns. One nurse recounted: “*this was a concern early on, now we’re going to be flooded with messages. And this did not turn out to be the case…”* (Staff Interviewee 1050). Another staff member emphasized: *“upfront it seems very overwhelming, but when you really start doing it and you start getting engaged, it’s so simple… it’s so helpful to see the patients’ symptoms throughout their recovery period”* (Staff Interviewee 1058)

Patients noted the eSyM interface in the EHR portal was easy to navigate, with few technological problems. Patients emphasized that the program’s integration into the portal was effective and fit seamlessly into their care experience with existing portal use. One patient commented: *“They send me an email saying check your portal, and it’s usually about twice a week that I get it. Then I just go in and fill out the eSyM, and…it’s simple. I don't know why more people don’t do it”* (Patient Interviewee 5). Staff reported similar perceptions of patient experience, having heard positive reviews during patient appointments.

#### Impact on patient-care team communication relationship

Patients and staff felt eSyM increased proactive symptom monitoring, fostering effective patient-care team communications. Nearly all patients expressed the view that eSyM’s symptom trajectory function, which allows visualization of past responses and symptom trends, was valuable. The ability to self-report symptoms added value for patients, who perceived closer connections with their care team. Many patients reported eSyM served as a “gut-check”; symptom questionnaires helped to increase comfort level and personal engagement in their care. One patient recalled: “*the doctor always mentions it, so I know that the doctors look at these, and so it gives me a – it gives me a source of like, okay, even when I’m not up there getting treatment and whatnot, somebody is watching out for what’s going on with me.”* They then further emphasized*: “…having the eSyM is a good way to kind of check on some of that. If I’m having a pain here or a pain there or whatever, it’s a good way to kind of put my mind at ease or they’ll either call me or they’ll – and they’ll ask me more questions, and it’s just kind of a good checkpoint for mental health, too”* (Patient Interviewee 5).

Staff members appreciated eSyM’s functionality, enabling understanding of baseline symptom burden, facilitating better patient connections, and the ability to contextualize symptom changes. Many expressed that symptom reports gave more holistic understandings of patients’ experiences between visits, especially for patients who were more independent in their care, enabling clinicians to better triage them. A nurse echoed: *“I think that it’s patient-centered and it puts us in better communication with our patients, gives them an opportunity to express themselves and to feel heard, and valued.”* They continued: *“I think it’s important, because it gives another avenue for patients to communicate how they’re feeling when they might not normally even call. It gives more information to a provider who has to make real-time decisions about the patient’s care. And being a nurse who assesses adverse events in patients all the time, I know that they can often under-share or feel more comfortable sharing some things electronically to a questionnaire than to a real person. So, I think we might capture things that we might not normally capture in a clinic visit because that patient feels more comfortable reporting it on a questionnaire”* (Staff Interviewee 12).

#### Although effective, ePROs are not top priority

While patients and staff found eSyM was easy to use, and a majority emphasized their views about the program’s potential, concern was expressed that competing priorities took precedence over engagement and symptom report responding. Because eSyM was launched as the COVID-19 pandemic began, care teams faced myriad challenges which lessened their ability to interact with eSyM. Staffing issues, including significant turnover and changes in hospital operations, diverted attention from eSyM implementation activities and delayed engagement while hospitals adjusted to unprecedented circumstances. While expressing an interest in and support for eSyM, staff also expressed regret that it was not initially as successful as hoped. Changes, including staffing shortages and high turnover, hindered involvement in the initial eSyM implementation. One staff member acknowledged that eSyM was superseded by competing priorities in the clinic: *“The priority was lessened in the face of COVID. For quite a while, the conversation was how to continue treatment safely. And so that was pretty much everyone’s bandwidth”* (Staff Interviewee 1005).

Patients also reported experiencing competing demands, namely that illness and family matters took precedence. Patients experiencing high levels of stress found it more difficult to engage with eSyM consistently, even if they found the program helpful. Similarly, patients experiencing a higher symptom burden reported being less likely to use eSyM and preferred more direct contact with care teams. One patient noted: *“I didn’t really use it. It was too long and drawn out. When you’re going through chemo, you don’t really feel like doing all that”* (Patient Interviewee 10). Patients who reported receiving poor prognoses or experiencing severe sickness also reported being less likely to use eSyM. Competing priorities significantly impacted the abilities of both cohorts to use eSyM.

### Differences in patient and care team experiences with eSyM

#### Symptom reporting expectations

Among most patients and staff, there was significant discordance in expectations after symptoms were reported via eSyM. Patients who reported severe symptoms received alerts to contact their care team, who also received a message indicating that a severe symptom had been reported. Patients often assumed their care team would immediately follow up after submission; however, care teams oftentimes did not respond to alerts promptly. One patient lamented that their sole complaint was a result of “…*my concern that there was no follow through…that’s my criticism.”* (Patient Interviewee 8).

Care teams frequently reviewed symptoms, noting that symptoms had been previously reported as chronic issues, and thus follow-up was not needed once addressed. One physician emphasized: *“these chronic issues…the providers don’t really need to know because they’re already aware of it”* (Staff Interviewee 19). Discrepancies in expectations about the timeliness of report follow-up between patients and care teams were consistently conveyed.

#### Perceived challenges to eSyM receptivity and technical use

Nearly all staff were initially apprehensive regarding patient willingness and ability to use eSyM. Many staff members expressed skepticism that older patients would be willing or able to meaningfully engage, noting that the technological components of the program may be too difficult or burdensome. However, patients’ willingness to embrace eSyM exceeded staff expectations. One doctor acknowledged: “*I was initially very apprehensive about patient receptivity, and I have been happily surprised by the patients’ positive response when I talk to them almost consistently. I was very worried about being able to explain procedures for the patients to access eSyM to get them on MyChart, if they weren’t yet on MyChart, and then to use the MyChart portal to locate the questionnaire and complete it. It went much better than I expected*” (Staff Interviewee 14).

All patient interviewees indicated a willingness to use eSyM because they believed it would increase their ability to self-manage symptoms at home, bolster their care involvement, and provide comfort knowing that providers were able to view symptoms between visits. Of note, no patients reported difficulty navigating eSyM. One patient commented: “*I thought it was pretty simple to see what you were requesting…I didn’t think it was very difficult at all to fill it out”* (Patient Interviewee 4).

## Discussion

In the setting of a type II hybrid effectiveness-implementation study of the novel, EHR-integrated eSyM program, a mixed-methods approach was used to assess its implementation across six health systems. Herein, we present the findings of the comprehensive qualitative analysis of 93 individuals with direct experience using eSyM in routine practice. We found distinct commonalities and differences in the experiences of staff and patients. Staff and patients believed eSyM was helpful, but both groups noted that competing demands hindered its use, perceiving that deeper user engagement is necessary for sustainability. Patients reported more willingness to use eSyM and fewer technical challenges with it than expected by staff. While staff initially reported concerns about the burden eSyM would have on workflow, the actual burden was modest. Focusing on facilitators and barriers to ePRO use in routine clinical practice, five key themes likely to be relevant to practices considering adoption of eSyM or similar programs were identified.

### eSyM has valuable clinical impact

Patients and staff reported overall positive experiences with eSyM, noting the intervention was easy to navigate and enabled symptom tracking. Most importantly, it served as a conduit to improve patient-provider communication, empowering patients regarding their symptom management needs. Our findings were like those of other research studies of remote symptom monitoring programs [24–26].

### EHR integration is key

Respondents noted that integrating the intervention into the EHR bolstered successful incorporation of eSyM duties into pre-existing routines. Patients could report outcomes through their institution’s portal in the same manner they would check appointments or messages. Similarly, clinicians could easily review reports alongside the rest of the patient’s chart. This is particularly important because effective symptom management relies on interdependence between patients and care teams; successful healthcare requires such co-productive partnerships [27]. Though concerns were initially expressed that patients would face technical challenges accessing eSyM, patients were often already familiar with portal usage; patient portal engagement across SIMPRO was >70% before eSyM deployment, making adoption relatively seamless [28].

### De-prioritization is a risk

Competing demands frequently deprioritized eSyM use by patients and staff. Some patients reported uncertainty about how important eSyM use should be in their daily lives. Since eSyM was deployed between 2019 and 2023, we hypothesize that the COVID-19 pandemic likely significantly altered the program’s implementation and uptake, perhaps reducing its priority, as has been shown in other healthcare-related work [29]. Employing recurring education and outreach strategies and distributing key performance indicators may help foster engagement, leading to more consistent and sustainable eSyM use.

### Misperceptions may have adversely impacted acceptability

One of the biggest barriers to eSyM uptake was the perception that eSyM would add insurmountable work to care teams’ list of responsibilities. As such, extensive efforts were made to embed eSyM into routine workflows, and training and outreach were used to promote long-term sustainability. Quantitative analyses demonstrated that severe symptom alerts were uncommon (<15% of reports), and calls to manage those alerts were even scarcer [30]. Misperceptions about program burden were challenging to overcome, and may have led to less frequent usage.

### Discordant expectations were common

Contrary to staff concerns, patients reported a strong willingness to use eSyM if they believed their clinicians were interested in the ePRO reports. Many patients reported they often did not receive calls from their care team after reporting severe symptoms, though they expected call-backs would be frequent. Care teams, however, used clinical discretion to decide whether to follow up promptly or wait for an upcoming appointment to discuss reports. This discordance left some patients resistant to subsequent ePRO submissions. When patients and care teams have discordant expectations, their relationship can suffer; increased education could help clarify patient and staff expectations regarding symptom report responsiveness.

## Limitations

First, qualitative data were anonymously collected, so feedback could not be linked with quantitative data (e.g., eSyM usage rates). Also, demographic information was not captured for staff, and only limited demographic information was captured for patients, so demographic characteristics were not reportable. However, participants came from six independent and geographically diverse health systems, so variability is expected. Second, the response rate and sample size for the patient subgroup was lower than expected, with only 15% (14/93) of approached patients completed interviews, which may have limited the representativeness of the patient sample. The challenge with patient accrual may have been due to the opt-in nature of the study, with email requests being the predominant method of patient outreach. That said, study leaders and qualitative experts agreed that meaning saturation was achieved after completing 14 patient and 79 staff interviews. Third, the overall sample size limited our ability to characterize the similarities and differences in perspectives amongst different patient or staff subgroups. Analyses by roles for staff and cancer type for patients could benefit future work and would be an important contribution to the literature. Fourth, participants who agreed to be interviewed are more likely to be knowledgeable of eSyM and could have more favorable opinions compared to those who declined to participate; selection and responder bias could have impacted the results. Fifth, confirmation bias could have emerged during interviews if participants sought to minimize negative feedback to appease interviewers, but this limitation was mitigated by obtaining a range of feedback from patients and staff at six diverse sites. Finally, variability in interview timing across sites (30–180 days for patients; 12–15 months for staff) may have influenced respondents’ recall and perception of the eSyM program.

## Conclusion

Evaluating the commonalities and differences in perception of eSyM amongst patients and staff who used the program as a component of routine clinical practice allowed for the identification of strategies to strengthen both the intervention and its implementation. Technological integration, workflow compatibility, and program impact were favorable attributes of eSyM. Competing demands, misperceptions about symptom alert burden, and discordant expectations may have adversely impacted dissemination. Our results may not be applicable in all healthcare settings; future studies in other settings and with different patient cohorts are warranted. For health systems looking to adopt and integrate eSyM or other ePRO systems into oncology practice, addressing these facilitators and barriers may help increase the likelihood of successful implementation, improving both the experience and outcomes of cancer care.

## Supplementary Information

Below is the link to the electronic supplementary material.

ESM 1 Supplementary Material 1 (DOCX 69.5 KB)

ESM 2 Supplementary Material 2 (DOCX 152 KB)

ESM 3 Supplementary Material 3 (DOCX 23.2 KB)

ESM 4 Supplementary Material 4 (DOCX 30.4 KB)

ESM 5 Supplementary Material 5 (DOCX 19.3 KB)

ESM 6 Supplementary Material 6 (DOCX 23.8 KB)

## Data Availability

In accordance with Cancer Moonshot grant guidelines, data are to be publicly available once published. An online link is forthcoming, but until that portal is live, please send all requests for data as written proposals to the corresponding author.

## Declarations

### Disclaimers

Dr. Wong conducted this work while a faculty member at Dartmouth Hitchcock Medical Center and Dartmouth Medical School.

Dr. Schrag conducted this work while a faculty member at Dana-Farber Cancer Institute and Harvard Medical School.

Dr. Dizon conducted this work while a faculty member at Brown University's Health Cancer Institute and Legoretta Cancer Center.
