# Supplementary material for: Patient and staff experiences with an EHR-Integrated Symptom Management Program (eSyM) in oncology
Source: Support Care Cancer. 2025 Dec 24;34(1):54. doi: 10.1007/s00520-025-10248-8 (PMC12738592; doi:10.1007/s00520-025-10248-8)
Supplement: Supplementary file 2 — Supplementary Material 2 (DOCX 152 KB) [file 520_2025_10248_MOESM2_ESM.docx]

**eSyM Patient Qualitative Interview Guide**

**[Introduction]**

Welcome! We have invited you today to participate in an interview with our team to share your experiences with the eSyM symptom management program. As you may recall, eSyM is a symptom management program offered to patients through MyChart (after beginning treatment or after discharge from surgery) that allows you to report your symptoms, access symptom tip sheets, and track your symptoms over time.

During our discussion today, we will be asking you questions and requesting your feedback to better understand your experiences with the program. Your responses will help us to find ways to improve this program and the symptom management process for future patients. Our goal is to help patients, caregivers, and their care team work together during and between visits to achieve better symptom control for patients. Your responses will not be shared with your health care team.

We anticipate that this interview will take about 1 hour to complete. To thank you for your time, we will send you a $15 Amazon.com gift card by mail or email when we are done, and as a reminder, you may choose to opt out or end this interview at any point.

Do you have any questions before we begin?

**[Demographic Questions]**

First, we will ask you a few questions about yourself.

1. What is your DOB?
2. What is your gender? [Male, Female, Other]
3. What is your race? [White, African American, Asian, Other]
4. Would you consider yourself Hispanic or Latino?
5. What is your marital status? [Single, Married, Divorced, Widowed, Significant Other, Other]
6. What is the highest level of school you have completed?
7. Are you currently employed?
   1. If yes, full-time vs. part-time?
   2. If no, retired, disabled, student, homemaker, unemployed, or other?

**[Interview Questions]**

*Now, we would like to ask you about your initial introduction to the eSyM program.*

1. Do you remember being introduced to eSyM?
   - If yes:
     - How did you first hear about eSyM? (potential secondary prompts: did you learn about it via a message delivered through the portal, a meeting with a person (teaching me about chemotherapy/prepping me for surgery), a telephone call with a member of the eSyM team, other…)
     - What made you interested in using the program initially? (potential secondary prompts– Did you think it would increase communication with your care team? Did you think eSyM would be a good way to track your symptoms? Did you use it because your care team told you to use it? What about eSyM appealed to you/made you want to use it?)
   - If no:
     - Have you ever used the eSyM program? If NO 🡪 Skip to question #27
2. Did you regularly use the patient portal prior to using eSyM?
   - If so, what was the most valuable part of the patient portal? How did you use it (e.g., to send messages to your care team, to view lab results, to pay bills)

*Next, we would like to learn more about your eSyM use.*

1. How did you answer the questionnaires? Via phone or tablet, or via computer?
   - Which was your preferred mode of response and why?
2. Were you able to easily navigate to the eSyM homepage and the questionnaires in the patient portal? How long did it take you to answer the questionnaire?
3. If not, what made it the most challenging for you?
4. Did you fill out the questionnaires yourself or did someone help you?
   - If so, then who helped you?
5. How many times did you respond to questionnaires from the eSyM program?
   - Did you use the program consistently whether you had symptoms or not? Or, did you only complete questionnaires when you had symptoms?
6. After completing a questionnaire, did you ever receive a message from the patient portal prompting you to contact your care team to discuss a symptom? If so, what did you do and why?
7. Did you ever receive a call from your care team who were reaching out to help manage a symptom reported via the eSyM program?
8. What fostered your continued use or lack of continued use?
9. What was the biggest barrier (if any) to your use of the eSyM program?
10. What did you think of the questionnaire itself? Did they provide an accurate reflection of the symptoms you experienced?
    - Was there information you wanted to relay, but found it hard to do via the questionnaire?
11. Did you utilize the symptom tip sheets? If so, did you find them helpful? Would something different be more helpful in managing mild or moderate symptoms?
12. If you responded to at least one symptom questionnaire, did you know that you could look back to see how your responses changed over time?
    - Did you use this feature and, if so, what did you think of it?

*Now, we would like to learn more about your care team’s engagement and assistance with your symptom management.*

1. Was eSyM mentioned by your provider during any appointments?
2. Did you feel that your care team was viewing and responding to your eSyM questionnaires and reported symptoms?
3. After reporting severe symptoms and being prompted to call your care team, how quickly were you able to get symptoms resolved/follow up on reported symptoms?

*The next few questions ask about what changes (if any) you would make to the questionnaire.*

1. Are there any symptoms you would add to the questionnaire?
2. Are there any questions you would change in the questionnaire?
3. Our original questionnaire asked for you to report symptoms in the last 7 days, but we switched it so that you only report symptoms in the last 24 hours. Which do you think is best for reporting your symptoms?
4. New symptom questionnaires are usually sent out every 3-4 days. Do you think this is a good cadence? Would you increase or reduce the frequency of the questionnaires?
5. Are there any other suggestions/changes you would make to the questionnaire?

*Final thoughts*

1. Overall, did you find the program helpful?
2. Have you ever completed questionnaires for other doctor/clinic visits – besides eSyM? If so, how would you compare them to eSyM?
3. Did you feel that your symptom management improved with eSyM?
4. How would you describe your overall care experience?
   - Did eSyM enhance or hinder that experience?
5. Did eSyM impact your ability to communicate with your care team? Improved it/hindered it/had no effect on it?

*Questions for Non-Responder Patients/Limited eSyM Experience*

1. Have you ever used the MyChart patient portal or Patient Gateway? If yes, what did you use it for (e.g., view lab results, message care team, pay bills)?
2. Do you find the patient portal to be helpful?
3. How do you typically contact your care team if you have questions?
4. Do you feel your symptom needs are well met between your appointments?
5. If you have heard of eSyM, why did you choose not to use it?
6. What, if anything, would make you more likely to use a program like eSyM?

Thank you so much for your time today!
