# Supplementary material for: Patient and staff experiences with an EHR-Integrated Symptom Management Program (eSyM) in oncology
Source: Support Care Cancer. 2025 Dec 24;34(1):54. doi: 10.1007/s00520-025-10248-8 (PMC12738592; doi:10.1007/s00520-025-10248-8)
Supplement: Supplementary file 3 — Supplementary Material 3 (DOCX 23.2 KB) [file 520_2025_10248_MOESM3_ESM.docx]

**SIMPRO – 1-Year Post-Implementation Interview Guide**

Greetings! We are speaking to you today to ask for your assistance with a very important task – we are requesting your opinions, suggestions, and feedback on the implementation of **eSyM** (our NCI-funded moonshot initiative to implement patient-reported outcomes into routine cancer care) so far.

We launched eSyM at [SITE] on [DATE]. We want to hear from you about how it’s going: we are asking for your input on the facilitators and barriers regarding the implementation of the eSyM.

**This interview will take about 20-30 minutes and I will be recording it. Are you ready to get started?**

**_____________________________________________________________________________________**

**I’d like to begin by asking you about the usage of ePROs in cancer care?**

- 1. What do your team members generally think about integrating ePROs into cancer care? *(Evidence Strength and Quality)*

**Now, let’s discuss collecting ePROs through eSyM specifically.**

- 1. How familiar are you with eSyM?
  2. What did you think of eSyM when you first heard about it? *(Evidence Strength & Quality)*
  3. Now that eSyM has gone live what should be changed to make eSyM work better? *(Adaptability)*
  4. Have you reviewed the: *(Design Quality & Packaging)*

| eSyM Component | NO | YES | If Yes then:  What is your view about the content? | If Yes then:  What is your view about the design/deployment? |
| --- | --- | --- | --- | --- |
| Symptom questions? | NO | YES |  |  |
| Training materials for patients? | NO | YES |  |  |
| Training materials for staff? | NO | YES |  |  |
| The population management dashboard? | NO | YES |  |  |
| The Symptom management “Tip sheets” | NO | YES |  |  |

- - - Probe, how could the XXXX design be improved?
  1. What factors have made it easier for your patients to use eSyM (if any)? Has any factor made it more difficult? If so, what has made it more difficult? *(Patient Needs & Resources)*
  2. Has eSyM impacted your workflow? If so, how? *(Compatibility)*
  3. Does eSyM make your work easier or harder? Can you explain? *(Compatibility)*
  4. What else could be done to engage patients? *(Engaging)*
  5. What else could be done to engage staff? *(Engaging)*
  6. Do you have a way to get the information you need about eSyM? *(Access to Knowledge and Information)*
  7. Do you have sufficient resources to implement and administer eSyM? (Probe on what is missing, which resources are critical, which are secondary, etc.”) *(Available Resources)*

**Next let’s discuss the process of launching eSyM at XXX.**

- 1. To what extent is My Chart/patient portal enrollment an institutional priority? *(Goals & Feedback)*
  2. To what extent is eSyM specifically an institutional priority? *(Goals & Feedback)*
  3. Is eSyM implementation aligned with your institution’s goals? If yes, how? If not, why not? *(Goals & Feedback)*
  4. To what extent might the eSyM implementation take a backseat to other high-priority initiatives going on now? *(Relative Priority)*
  5. Who are the key leaders who need to be on board to make eSyM implementation succeed at your institution? *(Opinion Leaders)* To what extent are they on board? *(Opinion Leaders)*
  6. Did any local, state, or national initiatives, policies, or guidelines influence eSyM implementation? If so, which ones? *(External Policies & Incentives)*
  7. How has the COVID19 pandemic influenced implementation of eSyM at your site?? *(Inner Setting, Outer Setting: Patient Needs and Resources)*
     - *Has it impacted the speed, scope, or timing of implementation? If so how?*
     - *Has it impacted whether eSyM is viewed as a priority? If so how?*
     - *Has it impacted leadership, staffing, or funding? If so how?*
     - *Has it impacted physician or patient involvement?  If so, how?*
  8. How has the COVID19 pandemic influenced attitudes towards eSyM at your site? *(Characteristics of Individuals)*
  9. Do you think COVID will affect the effectiveness of eSyM? How? *(External Policies & Incentives) (Probe on how patients communicate symptoms with care teams, how patients manage their symptoms, etc).*
  10. What factors have to be in place to increase the chances that eSyM implementation is a success? *(Implementation Climate)*

***With these last few minutes, I’d like you to reflect on the first year of eSyM at XXX.***

- 1. How do you think the eSyM implementation process has worked so far? *(Executing)*
- What has worked well/been effective *(Executing)*
- What hasn’t worked well or could be improved? *(Executing)*
  1. What is your evaluation of the value of eSyM at your site so far? *(Reflecting & Evaluating, Relative Advantage)*
  2. What does a site that is considering adopting eSyM need to know? *(Reflecting & Evaluating)*

**Is there anything else you would like to discuss today?**

**Thanks so much for your time!!!!**
