# Supplementary material for: Patient and staff experiences with an EHR-Integrated Symptom Management Program (eSyM) in oncology
Source: Support Care Cancer. 2025 Dec 24;34(1):54. doi: 10.1007/s00520-025-10248-8 (PMC12738592; doi:10.1007/s00520-025-10248-8)
Supplement: Supplementary file 4 — Supplementary Material 4 (DOCX 30.4 KB) [file 520_2025_10248_MOESM4_ESM.docx]

DPP Process Coding

Codes\\1. SIMPRO.ESym

| Name | Description |
| --- | --- |
| Barriers.Facilitators | - code in child code - Broad-scope barriers or facilitators with no applicable sub-categories - Experience/perception not defined/implied |
| Experienced facilitators.barriers | - experienced barriers/facilitators, actually experienced during esym implementation. Code in child codes when possible - Broad-scope barriers or facilitators with no applicable sub-categories - Defined or implied as experienced |
| E. Care team level | - code in child codes; includes buy in from care team ((CFIR: Process. B. Engaging.Attracting and involving appropriate individuals in the implementation and use of the intervention through a combined strategy of social marketing, education, role modeling, training, and other similar activities) - Clearly experienced b/f by care team   - Care team: nurses, APPs, PAs, MDs (non-research or clerical staff) |
| E. Time | - references to time as a barrier.facilitator; not enough time; DOES not include timing of launch. - Time as a b/f.   - Ex. “we do(n’t) have enough time to call eSyM patients” - NOT timing of launch |
| E.Provider level | - provider level, physicians, nurses, APP, - Experiences of nurses, doctors, and other providers (SWs, NPs, etc) |
| E.Staff level | - CRC, other staff, research staff - Experiences of broad staff including research, clerical, etc. |
| E.Training | - experiences with training; - Experiences of training patients or staff |
| E. External factors | - influences like payers, CMS, commercial payers, - Experienced influence of outside policies (ex. State laws), insurers, outside events |
| E. Institution.Site level | - factors at institution or site level; including leadership - Institutional events or policies with actual affect on eSyM deployment/usage - Can include leadership as well |
| E. Leadership level | - E. Leadership level - Experienced influence of PIs, institutional leadership, identified eSyM champions |
| E. other barriers.facilitators. nos | - other barriers.facilitators. not otherwise specified, code in child code when possible; all others code here. - Broad-scope barriers or facilitators not applicable to designated categories - Defined or implied as experienced |
| E. cost | - cost; financial barriers/facilitators experienced - Financial factors experienced (ex. “Expanding Epic to XX clinic would be too costly, so they can’t have eSyM”) |
| E. Timing of launch | - when site launched, e.g. being first or last, before or after another site or disease group - Impact of site launch time on uptake of program - Ex. “We deployed two days before COVID started…”, “Being the last site helped us avoid kinks like XXX” |
| E. Patient Level | - barriers facilitators identified at the patient level. how patients are able to engage or are prevented from engaging with the intervention (CFIR : Outer Setting. A. Needs & Resources of Those Served by the Organization; The extent to which patient needs, as well as barriers and facilitators to meet those needs are accurately known and prioritized by the organization) - Broad-scope patient experiences with definite language - “3/4 of my patients liked eSyM” |
| Careteam.communication | - Broad-scope interactions between patient and care team that cannot be subcategorized |
| Phone | - Communication between pt. and care team over the phone (calls, texting, etc.) |
| Pt. Portal | - Communication between pt. and care team using Patient Gateway/Portal |
| In.Person | - Communication between pt. and care team in person (at appointments, chemo teaches, etc.) |
| Motivations | - Things shared by patient that encouraged their use, but aren’t necessarily otherwise categorizable (want to help research, feel it would help their care, etc.) |
| E. Patient training | - references to experiences of patient training - Definite experienced pt training - “The patients who **had** eSyM walkthroughs with nursed used it much more often” |
| E. Technical.technology | - - references to technical barriers; technology, etc.   - Definite tech-related b/f   - “The program only worked on iPads because of a system update”, “The technology was easy to install at our site” |
| Confidence.competence | - - A patient’s perceived or experienced ability to use technology |
| Access | - - Pt’s ability to access tech (“I have spotty WiFi”, “I have access to a phone and a laptop”, etc.) |
| Qnr.use | - - The patient’s experienced use of questionnaires (“I used the questionnaires every time they came up”, “I only used questionnaires to track my really bad symptoms”) |
| E. Compatibility | - - aligned with current goals, compatibility with current goals (CFIR inner setting compatibility; The degree of tangible fit between meaning and values attached to the intervention by involved individuals,)   - How well the program is aligned with the goals of the team/institution, or with the patient’s life/goals   - “Right now we are really focused on implementing PROs at XXX”, “Our focus is really more on XXX than PROs” |
| Symptom.management | - - How the patients used the questionnaires to monitor/control their symptoms   - “I felt eSyM helped me keep track of my symptoms after my surgery”, etc. |
| E. Workflow | - specific reference to workflow; incorporating esym into existing workflow - How the program directly impacted (or did not impact) clinical workflow - “Our nurses have three more calls a week to make”, “Our workflow has remained status-quo” |
| E. Job Role | - *e.g. I am a nurse so this will help me with symptom management. - Experiences as related to interviewee-specific job - “As a nurse, this helped me better triage my weekly patient schedule”, “This only impacted oncs—as a surgeon I didn’t use it” |
| Intervention Materials | - reference to tip sheets, training materials, engagement materials - Tip sheets, training materials, engagement materials (NOT qnrs themselves) - “The tip sheets are really comprehensive”, “The training materials confused our nurses” |
| other barriers facilitators nos | - when unclear if it is perceived vs experienced; other barriers not otherwise specified that don;t fit in above codes. - Other things of note that were not clearly defined as experienced/perceived, or that don’t align with above codes |
| Perceived facilitators and barriers | - Code in Childcodes: (perception of regarding value; worth of esym) - Broad-scope barriers or facilitators with no applicable sub-categories - Defined or implied as perceived (no definitive language) |
| P. Institution level | - P. Institution level - Institutional events or policies with undefined effect on eSyM deployment/usage - Can include leadership as well |
| P.Leadership level | - P.Leadership level - Perceived influence of PIs, institutional leadership, identified eSyM champions - “I *think* if our PI was more involved it *may* have helped more people jump on the bandwagon” |
| P.Careteam Level | - code in child codes - Perceived experiences b/f by care team   - Care team: nurses, APPs, PAs, MDs (non-research or clerical staff) - “Our nurses seemed to like it, but I never asked them directly” |
| P.Provider level | - P.Provider level, physicians, APP, nurses - Perceived experiences of nurses, doctors, and other providers (SWs, NPs, etc) - “The social workers never looked like they got into it, I’m not sure if it would be useful to them” |
| P.Staff level | - P.Staff level- research staff, CRC, etc - Perceived experiences of broad staff including research, clerical, etc. - “The research team might have been better served doing XXX” |
| P.other barriers.facilitators nos | - other overall perceptions of the intervention - Other things of note that were clearly defined as perceived, but that don’t align with above codes |
| P.Patient level perceptions | - P.Patient level perceptions - Broad-scope patient experiences with indefinite language - “I think it would have been easier for patients to use it if XXX”” |
| Covid | - any reference to covid (CFIR: outer setting; covid) - COVID/the pandemic’s impact on implementation - “Since we had to go mostly online, more patients were comfortable with using new portal programs” |
| General Context | - Code in Child codes - Broad-scope items to contextualize f/b - “To let you know, I was out for three months at the start, so I don’t know how smoothly implementation was in the beginning” |
| Patient demographics.information | - Patient background that does not fit under other categories, but is important to understanding patient or cohort - “I am 53 and divorced”, “My husband is also undergoing treatment, so it makes it harder for me to monitor my own symptoms”, etc. |
| General Patient Care Experience | - General Patient Care Experience - General experiences of patients, not necessarily eSyM-related - “All of our patients get assigned a social worker when they enter our care” |
| Overall perception of ESym | - Overall perception of intervention; what do your team members generally think, big picture, about integrating electronic patient reported outcomes into cancer care; also overall views specific to ESym, level of familiarity w.esym, value of the system - Big picture thoughts specific to eSyM program - “I am very familiar with eSyM”, “I think this program is really valuable to our patients” |
| Overall perception of PRO | - overall perception of PROs (outside of ESym)l * when unclear ; place under Esym code - Big picture thoughts specific to PROs, but not just eSyM - “PROs are useful and all hospitals should be developing programs to collect them” |
| populations served | - population served by center - Pop-served by site - “We get patients from all the way in Kentucky!”, “The majority of our patients are elderly, Hispanic females” |
| type of health center | - type of health center e.g community, academic, urban, rural - Hospital/site category - “We are a high-volume clinic”, “We are in a really rural area, but we are an academic center” |
| NA | - Not applicable - “It’s really snowy here today; how about in Boston?” |
| Other NOS | - other ideas not otherwise specified/unclear if it pertains to Esym - Ideas that may/may not be applicable to eSyM - “Breast cancer clinics need to expand their research volume” |
| Suggestions.Recommendations | - Intervention Characteristics. D. Adaptability.Suggestions : . Suggestions for improvement can be captured in this code - Broad-scope intervention suggestions |
| Careteam Level suggestions | - Careteam Level suggestions (staff and provider) - Suggestions relating directly to provider - “Surgeons would probably use this more if you added XXX”, “Our nurses would like it if it didn’t alert X pool” |
| other suggestions recommendations | - Suggestions not specific to other categories - “Have you considered changing the name to something more catchy?” |
| Patient Level suggestions | - Patient Level suggestions - Suggestions relating to patient care or populations - “I think glioblastoma patients would find this really useful” |
| Technology Suggestions | - Technology Suggestions (includes questionnaires, 30-60-90 days suggestions references) - Suggestions relating to technology (questionnaires, look-backs, etc) - “Surgical patients should only have this for two weeks”, “I think you should add a text-box”, “I wonder if you made this its own app if more people would access it” |
