# Supplementary material for: Patient and staff experiences with an EHR-Integrated Symptom Management Program (eSyM) in oncology
Source: Support Care Cancer. 2025 Dec 24;34(1):54. doi: 10.1007/s00520-025-10248-8 (PMC12738592; doi:10.1007/s00520-025-10248-8)
Supplement: Supplementary file 5 — Supplementary Material 5 (DOCX 19.3 KB) [file 520_2025_10248_MOESM5_ESM.docx]

**Supplementary Material 5. Sample Interview Quotes Mapped to Thematic Domains: Differences in Patient and Staff eSyM Interactions**

| **Domain** | **Sub-Domain** | **Theme** | **Cohort** | **Interview #** | **Quote** |
| --- | --- | --- | --- | --- | --- |
| Differences | Symptom Reporting Expectations | Alerting and Chronic Symptom Responses | Patient | 6 | *“I didn’t even get the impression that anybody saw them, to tell you the truth… there was never any feedback, there was never any acknowledgement of receipt even. And you just got the sense that they were falling into a void someplace, you weren’t really sure anymore why you were answering them.”* |
|  |  |  | Patient | 11 | *“Knowing that someone is actually reviewing that to make sure that I’m okay I think would have mad me even more interested in filling it out… I was willing to fill it out for collecting data, but knowing someone actually really looks at those and contacts you if they see something wrong would, I think, help a lot of people.”* |
|  |  |  | Staff | 1002 | *“The patient was showing me, like, I've done these surveys, but it says you've never opened them. But in reality, every one of them have been opened and reviewed on our side, but it wasn't crossing over... So she's like, well, why would I continue to spend my time doing this if you're not even reading it?”* |
|  |  |  | Staff | 1019 | *“Right now, I don’t even think it impacts them at all because it’s more chronic issues that we don’t really have to follow up on.”* |
|  |  |  | Staff | 1034 | *“…if responding to eSyM reports by patients was part of a job description, there would be a motivation to follow up…”* |
|  |  |  | Staff | 1045 | *“If you have a patient that calls you three times a day already, then they took a questionnaire and you’re having to follow up… it could be a little redundant.”* |
|  |  |  | Staff | 1045 | *“If there’s no change in symptoms, we kind of just track and monitor”* |
|  | Perceived Challenges to eSyM Receptivity and Technical Use | Perceptions of Patient Willingness | Patient | 5 | *“I don't understand why more people don't use the eSyM because it's so simple but yet it can be so much help if you really need it.”* |
|  |  |  | Patient | 5 | *“It gives me a source of like, okay, even when I’m not up there getting treatment and whatnot, somebody is watching out for what’s going on with me. So, I find that very helpful.”* |
|  |  |  | Patient | 7 | *“It’s just nice to reflect, take a moment and say like, okay, this is how I’m feeling and I’m feeling however it is, whatever’s going on.”* |
|  |  |  | Patient | 13 | *“I mean I was more than willing to fill it out… but knowing that someone is actually reviewing that to make sure that I’m okay I think would have made me even more interested in filling it out… I think that would help a lot of people.”* |
|  |  |  | Staff | 1002 | *“I don't think it makes it easier by any stretch. Because it's another thing we teach in support, but it’s the right work being done. I'm just surprised at the lack of patient buy in that we've had.”* |
|  |  |  | Staff | 1006 | *“It takes us several tries to implement it. And even then, sometimes people would rather go back and do it a way that's objectively worse because of what they're more familiar with.”* |
|  |  |  | Staff | 1020 | *“I believe in it, and I think that it’s a way for us to innovate care delivery, but I think that it’s not the type of thing that’s woven into the fabric of our culture as an organization. We are not probably in a lot of ways prioritizing care delivery innovations in the same ways that we are scientific and translational innovations in the lab as an organization.”* |
|  |  | Perceptions of Technical Ability | Patient | 2 | *“It’s very easy… I just add it to my Google Calendar.”* |
|  |  |  | Patient | 3 | *“I do think it's a very informative and easy system, as far as I'm concerned”* |
|  |  |  | Patient | 14 | *“If people are going to use technology, they’re going to use technology… So to me, I’m high on technology, so it’s very easy to manage my health the way it’s going, so I love it.”* |
|  |  |  | Staff | 1001 | *“Our patients don’t automatically adapt, it requires an introduction and then ongoing reinforcement in their visit to get them comfortable using the tool.”* |
|  |  |  | Staff | 1007 | *“Just being able to read and understand written instructions. That’s definitely a barrier.”* |
|  |  |  | Staff | 1011 | *“The patients we would be hoping to reach, unfortunately, are patients that aren’t very engaged electronically in their care to begin with…”* |
|  |  |  | Staff | 1014 | *“The 30 percent or so who don’t have it usually they don’t – they’re totally computer – they don’t have a computer or they’re very tense about computers. And it’s hard for them to organize having a friend or a child come over to help them do the surveys. So they have their personal technological difficulties.”* |
|  |  |  | Staff | 1016 | *“A good chunk of the patients are really not technologically savvy, and a lot of them don't have smartphones, and are not savvy with that sort of stuff. So that's probably one thing.”* |
