# Supplementary material for: Patient and staff experiences with an EHR-Integrated Symptom Management Program (eSyM) in oncology
Source: Support Care Cancer. 2025 Dec 24;34(1):54. doi: 10.1007/s00520-025-10248-8 (PMC12738592; doi:10.1007/s00520-025-10248-8)
Supplement: Supplementary file 6 — Supplementary Material 6 (DOCX 23.8 KB) [file 520_2025_10248_MOESM6_ESM.docx]

**Supplementary Material 6. Sample Interview Quotes Mapped to Thematic Domains: Commonalities in Patient and Staff eSyM Interactions**

| **Domain** | **Sub-Domain** | **Theme** | **Cohort** | **Interview #** | **Sample Quotes** |
| --- | --- | --- | --- | --- | --- |
| Commonalities | Workflow Compatibility | Compatible with existing patient and clinical workflows | Patient | 4 | *"I get an email every time there's a new [eSyM]… so I always check it immediately, and I answer it immediately when it's in there."* |
|  |  |  | Patient | 7 | *"I had been writing things down about the way I felt, so this was just an easier message for me. Just thinking I should collect how I was feeling to get ready for my next visit, but then this came through and I was like, oh, this is probably what it’s for."* |
|  |  |  | Staff | 1004 | *"It’s an easy view, right at the beginning – at start of the day, we can see what patients that we need to target and provide additional education. Another part of my day is going through the report and reviewing those patients who have not completed a questionnaire in the past 14 days, in the past two weeks, to offer a friendly reminder and to extend my help in patients who have had any challenges of accessing, needing to reset their notifications and just giving them a courtesy call to help in any what that I can."* |
|  |  |  | Staff | 1015 | *"One of the, again, unexpected consequences that I learned with eSyM is, if you have a new nurse, eSyM makes it really much easier to understand, how do you get feedback from patients. And it imposes this sort of external, mild, moderate, severe paradigm with triggers, like, call your nurse, which didn't exist before or you really had to rely upon nursing judgment."* |
|  |  |  | Staff | 1050 | *"I think it is adding value. And the more they use it, the more they see the benefit of having it… I'm hearing in the clinic… how they've actually used the report from yesterday and help them take better care of the patients. So, they're incorporating it in their kind of daily activities."* |
|  |  | Technology compatibility due to portal integration and easy to use | Patient | 2 | *"It was really good. It was very easy – user-friendly. It was… a good program… I've been a nurse 33 years, so I was like, it's pretty catchy all around. You know, it's well rounded."* |
|  |  |  | Patient | 3 | *"It's just a form I fill out much like the other healthcare forms, so I don't really see any additional problems with your system over what I'm using on a daily basis, anyway."* |
|  |  |  | Patient | 3 | *"I do think it's a very informative and easy system, as far as I'm concerned."* |
|  |  |  | Patient | 5 | *"It just pops up and you just start answering the questions. It's… very easy to go through."* |
|  |  |  | Staff | 1017 | *"Yeah. I think the value has been good in the sense that it really engages the patient portal, the use of MyChart, the integration with rural health and the use of telemedicine. So, I think, globally, I think it's been really good."* |
|  |  |  | Staff | 1024 | *"So, I think the benefit of [eSyM] was that it was built into the system, which forced patients to at least acknowledge they had a portal and then went as far as to really encourage use of the portal."* |
|  | Impact on Patient-Care Team Relationship | Increased communication and proactivity with symptom monitoring | Patient | 5 | *"I just find it helpful because I like to let them know what's going on in my body just because I don't know if it's anything I need to worry about… you know what I mean?"* |
|  |  |  | Patient | 5 | *"I mean if you've got something going on, you report it to eSyM, and I mean I literally have gotten called the same day or always the next day. So that's always helpful because it's helpful guidance and it's also helpful as far as your anxiety level and whatnot."* |
|  |  |  | Patient | 7 | *"It really made me think about how I was feeling, and I have tendency to gloss over things and I caught myself maybe second guessing and saying, you are feeling something here so you probably should note it. And the frequency of them was good, too, for that as well, because maybe one day I was feeling it and one day I wasn’t."* |
|  |  |  | Patient | 9 | *"I think it’s a great little program, and I like that it happens a couple times a week, and it stays on top of it. I like that the doctors respond immediately, and it goes with my prognosis. Every second counts, so it’s an amazing program."* |
|  |  |  | Patient | 9 | *"Usually within a half a day I get a response from a doctor."* |
|  |  |  | Staff | 1020 | *"I thought that eSyM was really a way for us to be able to help people efficiently manage their day, sort of use this information as a way for people to be able to prioritize what they needed to do by the patients who were having the most issues and maybe take away some of the work that they were doing, because it was such a proactive concept."* |
|  |  |  | Staff | 1022 | *"I think that I, again, was very excited about engaging the patient and helping to get them to report their own experience sooner in the process."* |
|  |  |  | Staff | 1023 | *"I think it has huge potential. I mean, it’s what we need to be doing to improve outcomes. I think that it’s improved communication between patients and care team members. And I think it reflects a broader emphasis that the institute’s placing on patient reported outcomes, and patient quality of life during cancer treatment. So, I think it has high value."* |
|  |  | Closer connection between patients and care team | Patient | 1 | *"I think there's a certain comfort level that the patient is being tracked and if there was anything concerning in the questionnaire, someone would get back to me. So, in that sense, that was a good thing."* |
|  |  |  | Patient | 1 | *"I certainly would advocate for it and keep it going because it does make the patient feel connected to the hospital and to the care team, and it is – it's kind of like a security blanket."* |
|  |  |  | Patient | 5 | "It puts your mind at ease that somebody's watching this and somebody's keeping track of this and so you're not just kind of going from treatment to treatment and you don't know what's going on inside your body." |
|  |  |  | Staff | 1012 | *"I just thought it was an extra layer of communication that wouldn't normally be available to patients, especially patients that are undergoing such intense treatment. I think there needs to be another lifeline or another way that they feel like they can connect, that where they don't feel like they're intruding on their physician’s day or having to schedule another appointment. I think it's an approachable format for reaching out to one's provider, from the patient's perspective."* |
|  | Although Effective, ePROs are Not Top Priority | COVID-19 | Staff | 1001 | *"Of course, our priority is clinical staffing and we’ve had – the COVID-19 virus has certainly affected our staffing patterns. And so, as we’ve had staff out, we’ve had to reassign and re-prioritize tasks. And so I think if we had not gone through COVID-19, I think we would have progressed more quickly than we did."* |
|  |  |  | Staff | 1002 | *“To be really honest, we have had a ton of turnover with the pandemic. I’ve had people that have gone and done COVID – worked in COVID units… We've had, I think, more turnover this calendar year, than probably ever in the Cancer Center”* |
|  |  |  | Staff | 1008 | *“I think everyone’s priorities shifted to the emergency at hand. And although it might settle out in the next – in the coming months to be more of a priority once they see that they can have more intensive contact with less burden on the practice. So that’s always a hope.”* |
|  |  | Competing Demands | Patient | 3 | *"So much information's being dumped on a patient at that time after surgery, depending on when they would elect to discuss it – you know, its priority is pretty low."* |
|  |  |  | Staff | 1002 | *"There's just a lot of other things that are pressing things. So, we definitely, I think, need to figure out a way to kind of keep it more at the forefront for staffing, as far as participation and education."* |
|  |  |  | Staff | 1014 | *"They’re not talking specifically to patients about it, and I think that that has really been affected by competing priorities."* |
|  |  |  | Staff | 1020 | *"But I think the reality is that there are so many different priorities that we have as an organization that it’s got to be very impactful, and it’s got to be seen as very impactful to get to the top."* |
